# Performance of a PLK1‐based immune risk model for prognosis and treatment response prediction in breast cancer

**DOI:** 10.1002/cam4.5813

**Published:** 2023-03-23

**Authors:** Yan Chen, Yiqing You, Qiaoling Wu, Jing Wu, Shujing Lin, Yang Sun, Zhaolei Cui

**Affiliations:** ^1^ Laboratory of Biochemistry and Molecular Biology Research, Department of Clinical Laboratory Clinical Oncology School of Fujian Medical University, Fujian Cancer Hospital Fuzhou PR China; ^2^ Department of Gynecologic Oncology Clinical Oncology School of Fujian Medical University, Fujian Cancer Hospital Fuzhou PR China

**Keywords:** breast cancer (BRCA), immune infiltration, polo‐like kinase 1 (PLK1), prognosis, risk model

## Abstract

**Objective:**

Polo‐like kinase 1 (PLK1), a serine/threonine‐protein kinase, functions as a potent oncogene in the initiation and progression of tumor. The aim of this study is to assess potential correlations between PLK1 expression and immune infiltration in breast cancer (BRCA) and construct a PLK1‐based immune risk model applicable for prognosis and treatment response prediction in BRCA.

**Methods:**

We collected data on PLK1 gene expression in BRCA patients from The Cancer Genome Atlas (TCGA) database. Thereafter, we analyzed the associations of PLK1 expression with immune cell infiltration and immunomodulators, and established a prognostic risk model based on seven PLK1‐associated immunomodulator genes and a nomogram for survival prediction.

**Results:**

BRCA prognosis, clinical stage progression, and tumor classification were all shown to be substantially correlated with PLK1 expression. The PLK1 gene was significantly enriched in T cell and B cell receptors and molecules of the chemokine signaling pathways. Specifically, PLK1 expression was positively correlated with the CD8^+^ T cell and regulatory T cell (Tregs) activation and negatively correlated with M2 macrophage infiltration. The seven‐genes‐based risk model could serve as an independent prognostic factor of BRCA. The risk model was markedly correlated with the expression of programmed cell death protein 1/programmed cell death ligand 1 (PD‐1/PD‐L1; both *p* < 0.001) immune checkpoints, and tumor mutation burden (TMB). High‐ and low‐risk BRCA patients identified by the risk model responded differently to anti‐PD‐1 and/or anti‐CTLA4 therapy, as well as common chemotherapy drugs, like cisplatin, paclitaxel, and gemcitabine.

**Conclusion:**

This PLK1‐based immune risk model can effectively predict the prognosis and tumor progression of BRCA, identify gene mutations, and evaluate patient's response toward immunotherapy and chemotherapy regimens.

## Graphical abstract

PLK1 regulated the degree of immune cell (e.g., CD8+ T cells, Tregs, etc.) infiltration and immunomodulator expressions, thus participating in the tumor‐specific immune response. The PLK1‐based immune risk model could accurately identify immunotherapy‐or chemotherapy‐sensitive breast cancer patients, which is expected to act as a biomarker for personalized BC treatment.

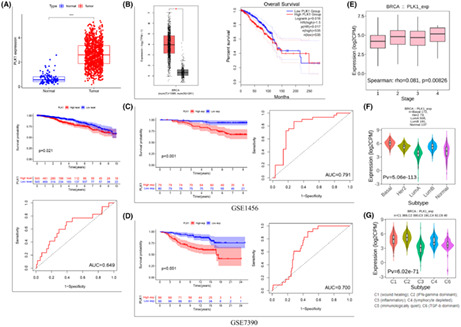

## 1 INTRODUCTION

Breast cancer (BRCA) is one of the most prevalent types of cancer, with the second‐highest morbidity and the fourth highest mortality across all human cancers.^1^ Currently, surgery is the preferred mode of treatment for majority of BRCA cases, followed by a combination of adjuvant or neoadjuvant therapy and radiotherapy.^2^ However, patients with distinct subtypes and pathological characteristics require a more flexible treatment regime. For example, patients are often treated with endocrine therapy or combined chemotherapy when the tumor is estrogen receptor/progesterone receptor (ER/PR)‐positive or treated with human epidermal growth factor receptor 2‐(HER2) targeted drugs (trastuzumab or pertuzumab) or concurrent chemotherapy when the tumor is HER2 positive. If the tumor is both HER2‐ and ER/PR‐positive, endocrine therapy is given separately. Chemotherapy is preferred for patients with triple‐negative breast cancer (TNBC), which is a more aggressive subtype of BRCA, exhibiting early onset, rapid clinical progression, and a poor prognosis.^3^ The median survival time of metastatic TNBC patients is only 10–13 months.^2^ Multimodal combination systemic therapy has shown remarkable efficacy in BRCA. Among these, immunotherapy has achieved the most extraordinary response and produced revolutionary outcomes in the treatment for various solid tumors, particularly improving the overall survival (OS) of patients.^4^, ^5^, ^6^ Studies have demonstrated that the OS of TNBC patients with positive programmed cell death ligand 1 (PD‐L1) could be prolonged following a single use of programmed cell death protein 1 (PD‐1)/PD‐L1 inhibitors, such as atezolizumab or pembrolizumab, with a higher efficacy achieved by a combination of chemotherapy and targeted drugs.^7^, ^8^ However, immunotherapy is not recommended for all BRCA patients due to different tumor immune microenvironment (TIME) conditions. Therefore, there is an urgent need for robust indicators of BRCA for prognosis and response prediction for efficient treatment of BRCA patients.

Polo‐like kinase 1 (PLK1), a serine/threonine‐protein kinase, serves as a regulator of mitotic G2/M progression, centrosome maturation, spindle assembly, cell cycle, chromosome segregation, DNA replication, cytokinesis, and meiosis.^9^ In addition, PLK1 expression, which is observed in various malignant tumors, often suggests poor prognosis.^10^, ^11^ The latest evidence implies an oncogenic role of PLK overexpression in cancer, resulting in defects in mitosis, cytokinesis, centrosome, and cell cycle checkpoints or inducing a persistent increase in chromosome instability, culminating in aneuploidy and tumor formation.^12^ Meanwhile, it has been shown that PLK1 stimulates tumor cell proliferation, inhibits apoptosis, and promotes the epithelial‐mesenchymal transition (EMT).^13^, ^14^, ^15^ Moreover, PLK1 overexpression has been associated with chemoresistance, which can reduce the efficacy of chemotherapeutic agents.^16^, ^17^ Therefore, PLK1 is a critical target for cancer treatment.^18^ PLK1 also controls the nuclear factor kappa‐light‐chain‐enhancer of activated B cells (NF‐κB) and interferon regulatory factor 3 signaling pathways, indicating that it may play a role in cellular immunity and inflammatory signaling, thereby influencing immune infiltration in the tumor microenvironment (TME).^13^ However, the association between PLK1 and TME is poorly known and requires further investigation. In conclusion, PLK1 may not only regulate tumor development, growth, and metastasis, but may also have substantial immunological implications in tumors.

Associations between PLK1 and TIME in all cancer types have not been thoroughly studied. In this study, we assessed PLK1 expression and its association with immune cell infiltration and immune regulatory factors in BRCA and developed a risk model based on our screening of PLK1‐related immunomodulator hub genes for BRCA progression. Additionally, we assessed the predictive performance of this model by assessing its correlation with clinicopathological factors and the gene mutation spectrum of patients and subsequently constructed a nomogram. Furthermore, we evaluated the efficacy of the model in immunotherapy or chemotherapy response prediction, and our results revealed that this model has a high predictive value for determining treatment regimes and can be implemented for personalized prescription for BRCA patients.

## 2 METHODS

### 2.1 Data acquisition and differential and survival analysis of PLK1 expression in BRCA

RNA‐seq, clinical, and gene mutation data associated with BRCA were obtained from The Cancer Genome Atlas (TCGA) (https://portal.Gdc.Cancer.Gov/). The RNA‐seq data consisted of PLK1 gene expression data from 1109 BRCA and 113 normal tissue samples. Clinical data, including survival time, survival status, age, gender, clinical stage, and TMN stage, were collected from 1097 BRCA patients. Gene mutation data included single‐nucleotide mutation of genes in 980 patient samples.

BRCA and control breast tissues were compared for differences in PLK1 gene expression. Kaplan–Meier (KM) survival curves and receiver operating characteristic (ROC) curves were plotted in conjunction with the survival data to explore the prognostic significance. Simultaneously, the results were reconfirmed by online differential and survival analysis of PLK1 expression in BRCA using Gene Expression Profiling Interactive Analysis 2 (GEPIA2; http://gepia2.cancer‐pku.cn/#index). Additionally, survival analysis of PLK1 expression was utilized to confirm the aforementioned findings in the GSE1456 and GSE7390 datasets.

### 2.2 Identification of co‐expressed genes associated with PLK1

Data on genome sequencing, chromosome copy number, and large‐scale parallel sequencing were collected from 1019 human cancer cell lines from the Cancer Cell Line Encyclopedia (CCLE) (https://portals.BroadInstitute.Org/ccle/data). Using CCLE‐BRCA RNA‐seq data, we searched for genes that were substantially co‐expressed with PLK1 (correlation coefficient [Cor] >0.5 and *p* < 0.001). Enrichment analysis of Gene Ontology (GO) and Kyoto Encyclopedia of Genes and Genomes (KEGG) was performed using clusterProfiler and pathview software package in R to elucidate the molecular mechanisms of the highly co‐expressed genes associated with BRCA development or progression (*p* < 0.05). Gene Set Enrichment Analysis (GSEA) was utilized to uncover signaling pathways associated with abnormal PLK1 expression in BRCA.

### 2.3 Identification of tumor‐infiltrating lymphocytes in BRCA

The cell‐type identification by estimating relative subsets of RNA transcripts (CIBERSORT) algorithm was used to describe immune cell infiltration by calculating the proportion of each subpopulation of the 22 immune cells in BRCA compared to the normal breast tissue samples,^19^ with the filtering standard set at *p* < 0.05. For each immune cell type, the RNA‐seq data was transformed into a score using the CIBERSORT method. A *p*‐value of ≥0.05 was considered statistically insignificant. Lastly, for the purpose of identifying tumor‐infiltrating lymphocytes (TILs) in BRCA, 727 BRCA and 70 normal breast tissue samples were analyzed. Additionally, we compared the amount of immune cell infiltration in various PLK1 expression groups.

From the 28 TILs, those associated with PLK1 expression in BRCA were confirmed by correlation analysis using TISIDB (http://cis.hku.hk/TISIDB/index.php), a portal website for interactions between tumor and immune system. Additionally, the data on clinical stage, immunophenotype, and molecular typing associated with PLK1 expression were acquired. TIMER2.0 (http://timer.comp‐genomics.org), a resource portal that systematically analyzes immune infiltration in pan‐carcinomas, was used to assess the associations of PLK1 gene copy number variations (i.e., normal diploid, arm deletion, arm amplification, and high amplification) with the selected TILs (e.g., CD4^+^, CD8^+^ T cells, and B cells) in BRCA.

### 2.4 Correlations between PLK1 and immunomodulators

We initially selected 42 immunostimulators and 21 immunoinhibitors from the TISIDB and assessed their associations with PLK1 expression in BRCA. GO and KEGG enrichment analysis was performed for PLK1‐associated immunomodulators to determine their roles in oncogenetic mechanisms associated with PLK1 in BRCA using WebGestalt (http://www.webgestalt.org/; an online portal for enrichment analysis), with the FDR <0.05. Search Tool for the Retrieval of Interacting Genes/Proteins (STRING; https://string‐db.org/) was used to resolve interactions between the selected immunomodulators and PLK1.

### 2.5 Establishment of a PLK1‐based immune risk model

Univariate Cox regression analysis was performed on PLK1‐related immunomodulators (*p* < 0.05) to screen prognostic genes. Variables with statistical significance were subjected to multivariate Cox regression analysis to determine whether the PLK1‐based immune model or the risk score were more powerful in prognosis prediction. BRCA patients were categorized into a high‐ or low‐risk group according to the median risk score of each patient, and KM survival curves and risk curves were drawn. The PLK1‐based immune risk model was confirmed using the GSE58812 dataset. We also conducted an infiltrating duct carcinoma and a lobular carcinoma KM survival analysis for distinct pathological types. In addition, correlation analysis was carried out to describe correlations between the model and clinical features, and the independence of this model as a robust predictor for BRCA patients was assessed using the univariate and multivariate CoX regression analysis. ROC curves were drawn to determine the prediction accuracy of the model. Finally, gene mutations in BRCA and tumor mutation burden (TMB) in patients were systematically evaluated. Additionally, the survival difference between the high‐ and low‐TMB groups was analyzed.

### 2.6 Construction of a nomogram of prognosis prediction

A nomogram for BRCA prediction was constructed based on regression analysis of the model and clinical features using the rms package in R, and the corresponding correction curves were plotted. Each predictive index was scored according to its contribution degree to OS. The OS rate of patients was calculated based on the sum of each index score (or the total score). The correction curve depicting a difference between the actual and predicted risk was plotted to determine the prediction accuracy of the nomogram.

### 2.7 Response assessment of immunotherapy and chemotherapy for BRCA using the PLK1‐based immune risk model

We first identified immune checkpoints that were significantly associated with the risk model from the eight common immune checkpoints in BRCA, PD‐1, PD‐L1, PD‐L2, cytotoxic T‐lymphocyte associated protein 4 (CTLA4), lymphocyte activating 3 gene (LAG3), TIM‐3, T cell immunoreceptor with Ig and ITIM domains (TIGIT), and lemur tyrosine kinase 3 (LMTK3), to determine the patients' immune status. To determine the patients' response to immunotherapy, the relative immune genome analysis results of next‐generation sequencing analysis and immunophenoscores of BRCA patients were available in The Cancer Immunome Atlas database (https://tcia.at/home). The potential difference in response to immunotherapy was predicted between patient groups stratified using the risk model. Lastly, the pRRophetic package in R was employed to predict the drug sensitivity of BRCA patients to four first‐line chemotherapy drugs,^20^ paclitaxel, cisplatin, doxorubicin, and gemcitabine, based on the 50% concentration inhibition (IC_50_) values.

### 2.8 Statistical analysis

Commercially available R (version 4.0.2) and GSEA (version 4.1.0) software were used for all statistical analyses. Survival differences between high‐ and low‐risk BRCA groups were assessed using the Log‐rank test. Continuous variables were evaluated using the Wilcoxon test. The chi‐square test was utilized for discontinuous variables. Spearman's rank correlation test was employed for all the correlation analyses. All tests were two‐tailed tests and a *p*‐value of <0.05 was regarded as statistically significant.

## 3 RESULTS

### 3.1 PLK1 gene expression in BRCA tissues and several BRCA subtypes

**FIGURE 1:**
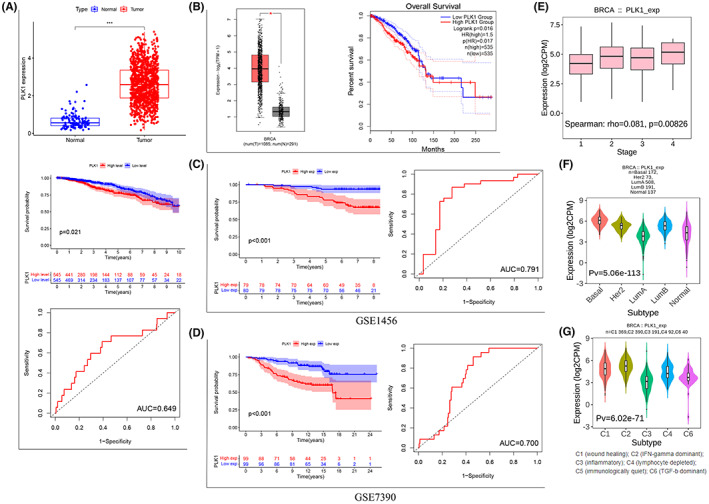
Comprehensive analysis of polo‐like kinase 1 (PLK1) expression in breast cancer (BRCA) and its subtypes. (A) Differential expression analysis of PLK1 between BRCA and normal breast tissues, Kaplan–Meier curve survival analysis, and time‐dependent ROC curves were performed using the data obtained from The Cancer Genome Atlas (TCGA). (B) The differential PLK1 expression and the survival prediction capability of PLK1 were validated using Gene Expression Profiling Interactive Analysis 2 (GEPIA2). (C, D) The Kaplan–Meier curve survival analysis and receiver operating characteristic curves were performed for GSE1456 and GSE7390 datasets. The histograms and violin plots show (E) the clinical stage, (F) BRCA immune subtypes, and (G) molecular subtypes correlated with PLK1 expression in BRCA. *p < 0.05; ***p < 0.001.

Using TCGA data, we initially discovered that PLK1 expression differed between the BRCA and normal breast tissue samples, with the PLK1 expression much lower in the normal tissues compared to the BRCA tissues (*p* < 0.001). KM survival analysis revealed a shorter survival time in the high PLK1 expression group versus the low PLK1 expression group (*p* = 0.021, *n* = 1097). Furthermore, the ROC curves suggested a moderate accuracy of PLK1 gene expression in predicting BRCA prognosis (AUC = 0.649; Figure 1A). GEPIA2 analysis supported PLK1 upregulation in BRCA and poor survival in PLK1 overexpression cases (Figure 1B). These findings were further confirmed in the GSE1456 and GSE7390 datasets, indicating consistent survival (*p* < 0.001) and strong predictive effectiveness (AUC = 0.791, 0.700; Figure 1C,D). Furthermore, there was a significant difference in PLK1 expression between different clinical stages and BRCA subtypes (Figure 1E–G). PLK1 expression levels were significantly higher in stage II, III, and IV patients than in stage I patients, and the most significant increase was observed in stage IV patients (*p* = 0.00826). PLK1 expression was exceptionally high in HER2‐positive and luminal B‐like, particularly basal‐like, BRCA samples compared to the normal control samples. Usually, basal‐like BRCA is TNBC because they are highly overlapping and have a poor prognosis.^21^ In addition, PLK1 expression was significantly correlated with immune subtypes, with the lowest expression in the C3 subtype and the highest expression in the C2 subtype.

### 3.2 Co‐expression genes of PLK1 in BRCA

**FIGURE 2:**
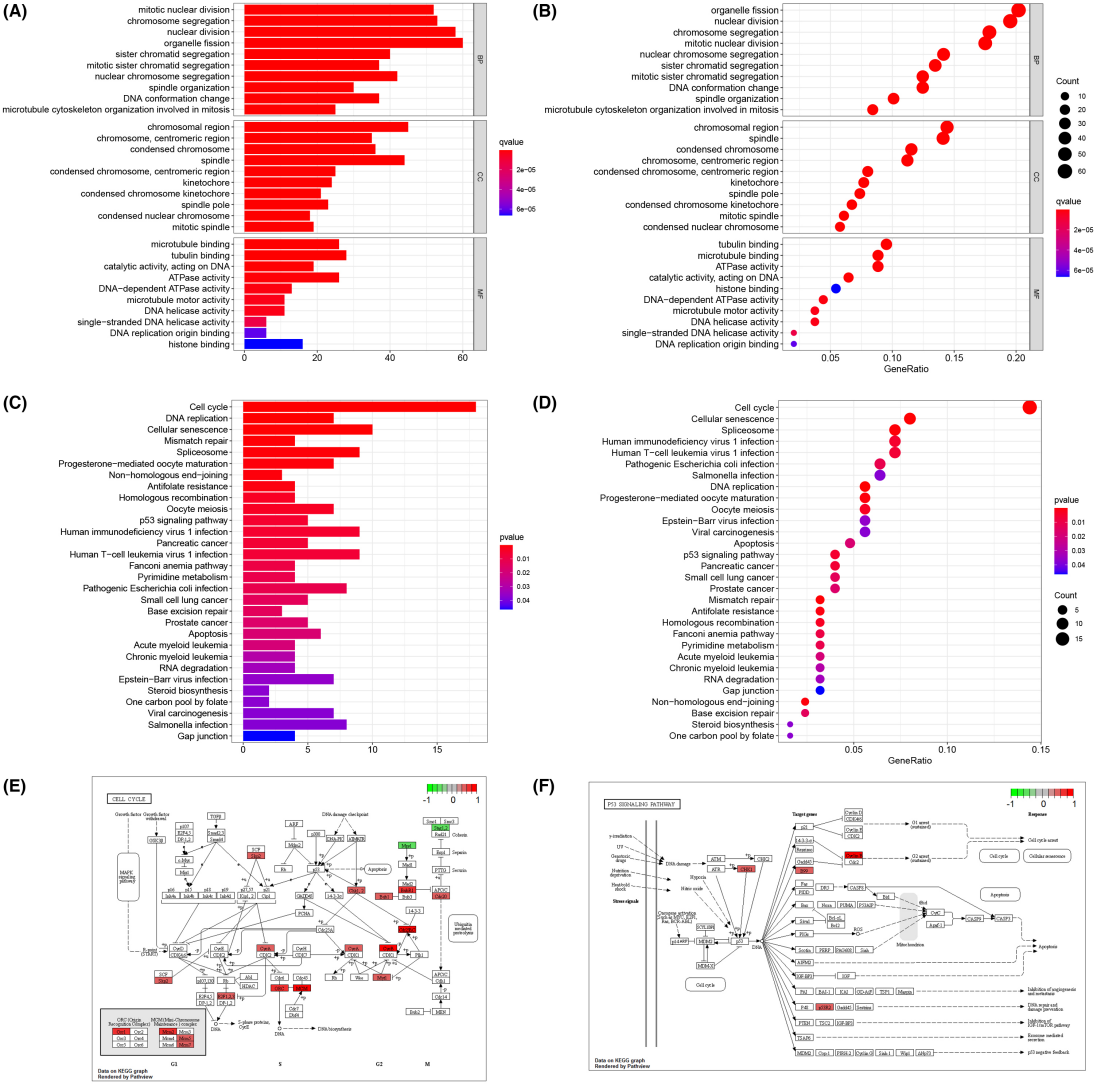
Analysis of Gene Ontology (GO) and Kyoto Encyclopedia of Genes and Genomes (KEGG) enrichment for genes co‐expressed with polo‐like kinase 1 (PLK1). (A, B) The histograms and bubble charts for GO enrichment analysis and (C, D) KEGG enrichment analysis. The *x*‐axis of the histogram represents the number of enriched genes, while the color scale from blue to red represents the degree of enrichment. The *x*‐axis of the bubble chart indicates the percentage of genes that have been enriched. The size of the bubble indicates the number of enriched genes, and the color scale from blue to red indicates the degree of enrichment. (E, F) The correlation between the PLK1 co‐expressed genes and the cell cycle and P53 signaling pathway molecules. Negative and positive correlations are represented by green and red areas, respectively. A deeper shade suggests a stronger association.

We analyzed potential correlations between the RNA‐seq data of 51 BRCA cell lines, obtained from the CCLE database, with PLK1 expression, and initially identified 348 genes that were highly co‐expressed with PLK1. GO enrichment analysis of these genes revealed that under biological functions the genes were associated with organelle fission, nuclear fission, and chromosome segregation; under cell components the genes were enriched in chromosomal regions and spindle apparatus; and under molecular functions the genes were associated with tubulin binding and ATPase activity (Figure 2A,B). The KEGG enrichment analysis of the co‐expressed genes revealed that they are may be associated with cell cycle, cellular senescence, and P53 signaling pathways in BRCA (Figure 2C,D). The results of enrichment analysis supported the regulatory role of PLK1 as a serine/threonine‐protein kinase at the M phase of cell cycle, indicating that the co‐expressed genes were actively involved in oncogenetic role of PLK1 in BRCA. In addition, the co‐expressed genes regulating cell cycles were highly positively correlated with Cyclin B (CycB), cell division cycle 25 B/C (Cdc25B/C), MCM, and origin recognition complex subunit 1 expressions levels, and negatively correlated with Mpsl, stromal antigen 1 (Stag1), and Stag2 (Figure 2E). Additionally, the co‐expressed genes were positively correlated with CycB, G2, and S‐phase expressed 1 (also named B99), P53R2, and protein kinase Chk1 (CHK1) that are involved in the P53 signaling pathway (Figure 2F).

### 3.3 Association of PLK1 expression and immune cell infiltration in BRCA

**FIGURE 3:**
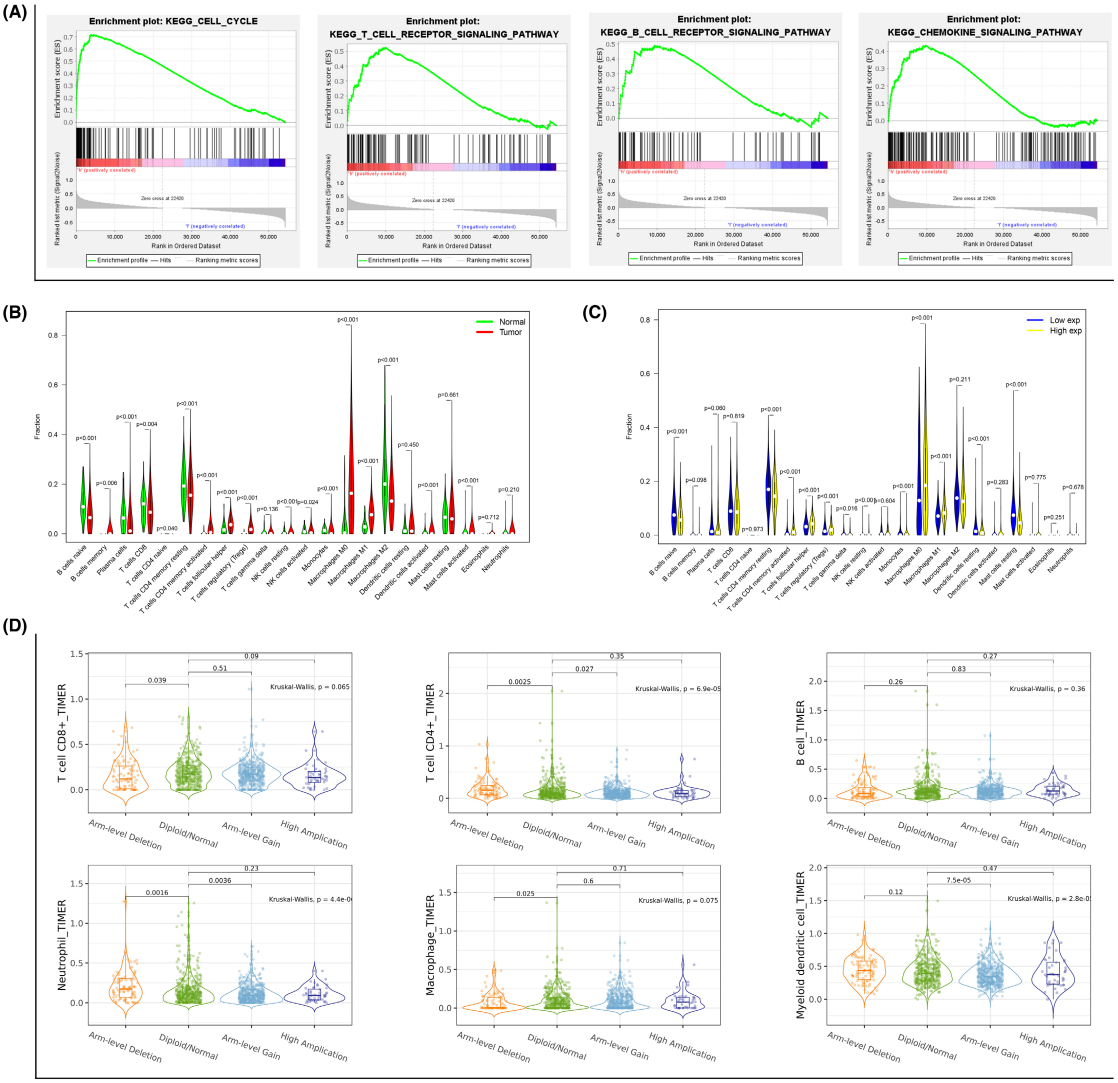
Gene Set Enrichment Analysis (GSEA) of polo‐like kinase 1 (PLK1) and immune cell infiltration in breast cancer (BRCA). (A) GSEA enrichment analysis of PLK1 and correlations of PLK1 with cell cycle and immune‐related signaling pathways. (B) The cell‐type identification by estimating relative subsets of RNA transcripts algorithm was used to assess the difference in the distribution of the infiltrating immune cells between BRCA and normal breast tissues. (C) Immune cell infiltration was compared between high and low PLK1 expression groups. (D) Violin plots demonstrate the correlations between PLK1 copy number variations and the infiltration degree of the six immune cells (CD8^+^ T cell, CD4^+^ T cell, B cell, neutrophil, macrophage, and myeloid dendritic cell).

GSEA enrichment revealed that PLK1 not only disturbed cell cycle (NES = 2.23, *p* < 0.001), but affected various immune‐related signaling pathways, including the T‐ (NES = 1.84, *p* < 0.001) and B‐cell receptor signaling pathways (NES = 1.75, *p* < 0.001). Among others, the chemokine signaling pathway (NES = 1.60, *p* < 0.001) was particularly essential (Figure 3A) as it implied the role of PLK1 in the immune regulation of BRCA. Additionally, compared with the normal breast tissues, we found significant increases in the frequency of memory B cells, activated memory CD4^+^ T cells, regulatory T cells (Tregs), M0 and M1 macrophages, activated dendritic cells, and activated mast cells (the primary tumor‐infiltrating immune cells in BRCA), and pronounced decreases in the frequency of CD8^+^ T cells, activated NK cells, and macrophages M2 (Figure 3B). We also analyzed the differences in the level of immune cell infiltration between different PLK1 expression profiles and found differences in 12 immune cells such as Tregs, activated memory CD4^+^ T cells (Figure 3C). Moreover, the degree of CD8^+^ T cell, CD4^+^ T cell, neutrophil, macrophage, and myeloid dendritic cell infiltration was significantly associated with the copy number variations of PLK1 in BRCA (Figure 3D).

**FIGURE 4:**
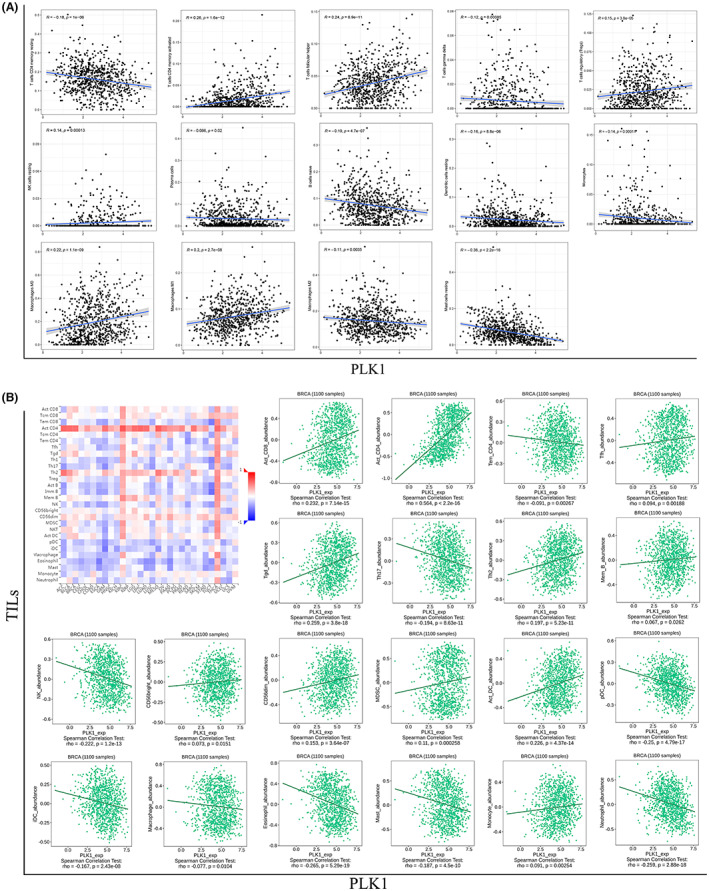
Relationship between polo‐like kinase 1 (PLK1) expression and immune cell infiltration in breast cancer (BRCA). (A) Correlation analysis between PLK1 expression and BRCA‐infiltrated immune cells using The Cancer Genome Atlas data. (B) Correlation analysis between PLK1 expression and tumor‐infiltrating lymphocytes (TILs) in the TISIDB database. The thermogram demonstrates the positively (red color) or negatively (blue color) correlated TILs with differential PLK1 expression in BRCA. A darker color indicates a higher correlation degree. The scatterplot reveals the correlation between PLK1 expression and TILs in BRCA.

In addition, the correlation analysis highlighted 14 tumor‐infiltrating immune cells associated with PLK1 expression in BRCA (Figure 4A), with six (Tregs, resting NK cells, activated memory CD4^+^ T cells, follicular helper T cells [Tfh], M0 macrophages, and M1 macrophages) showing enhanced tumor infiltration and eight (naive B cells, plasma cells, resting memory CD4^+^ T cells, gammadelta T cells, monocytes, resting dendritic cells, M2 macrophages, and resting mast cells) exhibiting diminished infiltration. The results from the correlation analysis between PLK1 expression and 28 TILs using the TISIDB database were nearly consistent with the above findings (Figure 4B), demonstrating that PLK1 expression was involved in regulating immune cell infiltration during the occurrence and development of BRCA.

### 3.4 PLK1 regulates the immunomodulator network in BRCA

**FIGURE 5:**
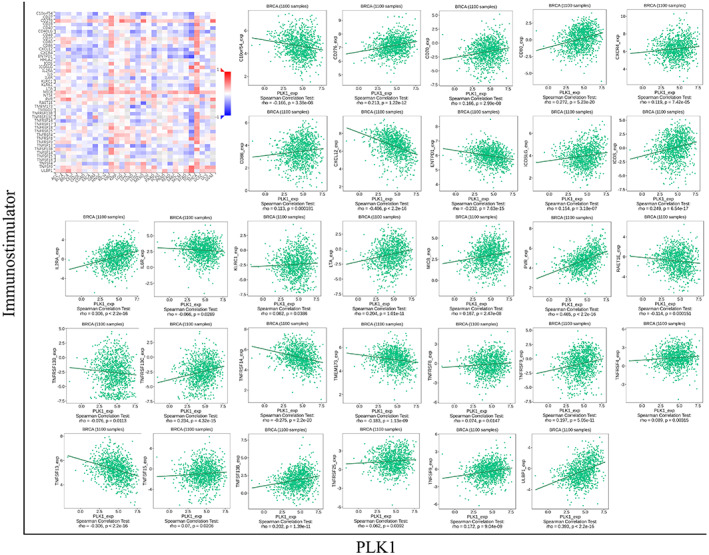
The immunostimulators associated with aberrant polo‐like kinase 1 (PLK1) expression in breast cancer (BRCA). The thermogram identifies the immunomodulatory factors that are positively (red color) or negatively (blue color) correlated with PLK1 expression in pan‐carcinomas. A darker color indicates a higher correlation degree. The scatterplot reveals 30 immunostimulatory factors associated with PLK1 upregulation in BRCA.

**FIGURE 6:**
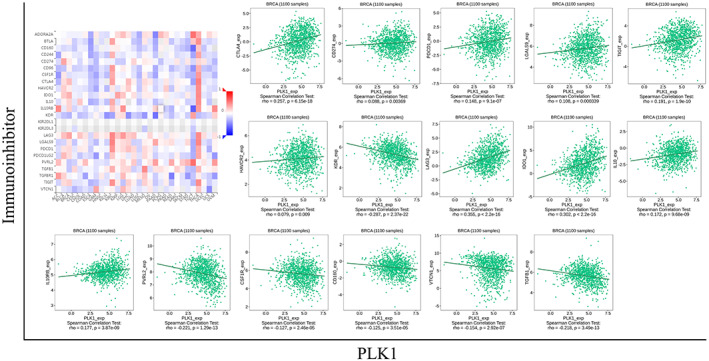
The immunoinhibitors associated with aberrant polo‐like kinase 1 (PLK1) expression in breast cancer (BRCA). The thermogram identifies the immunomodulatory factors that are positively (red color) or negatively (blue color) correlated with PLK1 expression in pan‐carcinomas. A darker color indicates a higher correlation degree. The scatterplot reveals 16 immunosuppressive factors associated with PLK1 upregulation in BRCA.

**FIGURE 7:**
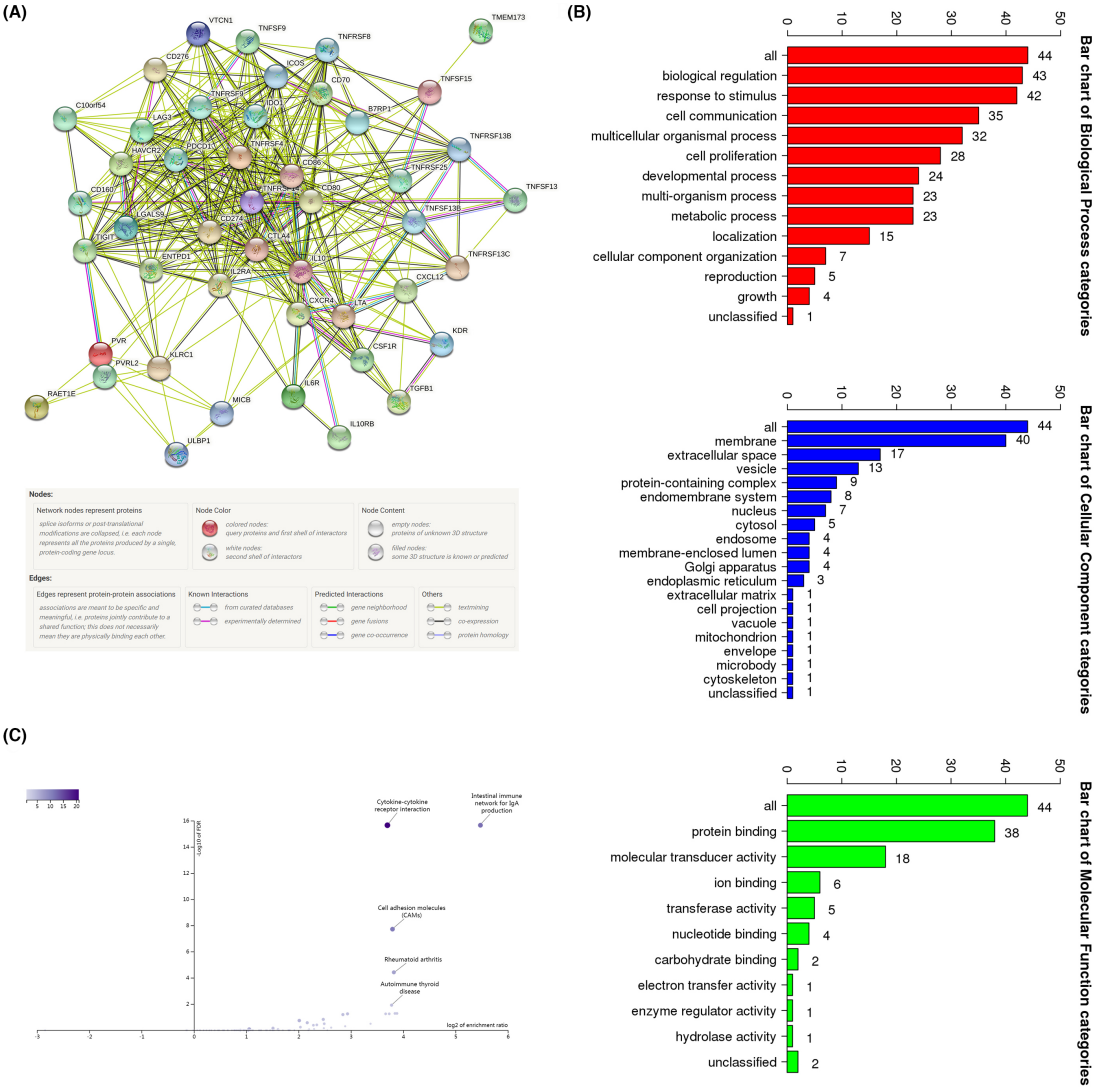
Polo‐like kinase 1 (PLK1)‐associated immunomodulatory factors enriched in Gene Ontology (GO) and Kyoto Encyclopedia of Genes and Genomes (KEGG). (A) Search Tool for the Retrieval of Interacting Genes/Proteins (STRING) protein–protein interaction network includes 46 PLK1‐associated immunomodulatory factors. (B, C) GO and KEGG enrichment analysis of PLK1‐associated immunomodulators. PLK1 may participate in response to stimulis, protein binding, and cytokine–cytokine receptor interactions via modulating these immunomodulators.

Forty‐six immunomodulators associated with PLK1 expression in BRCA were obtained from the TISIDB database, including 30 immunostimulators, such as CD80 and CD8, and 16 immunoinhibitors, such as CD274, CTLA4, and IL10 (Figures 5 and 6). Protein–protein interaction (PPI) networks were plotted for the 46 immunomodulators (Figure 7A). The GO and KEGG enrichment analysis indicated that PLK1 might participate in response to stimulus, protein binding, and cytokine–cytokine receptor interactions (Figure 7B,C) via regulating these immunomodulators.

### 3.5 Establishment of a PLK1‐based immune risk model

**FIGURE 8:**
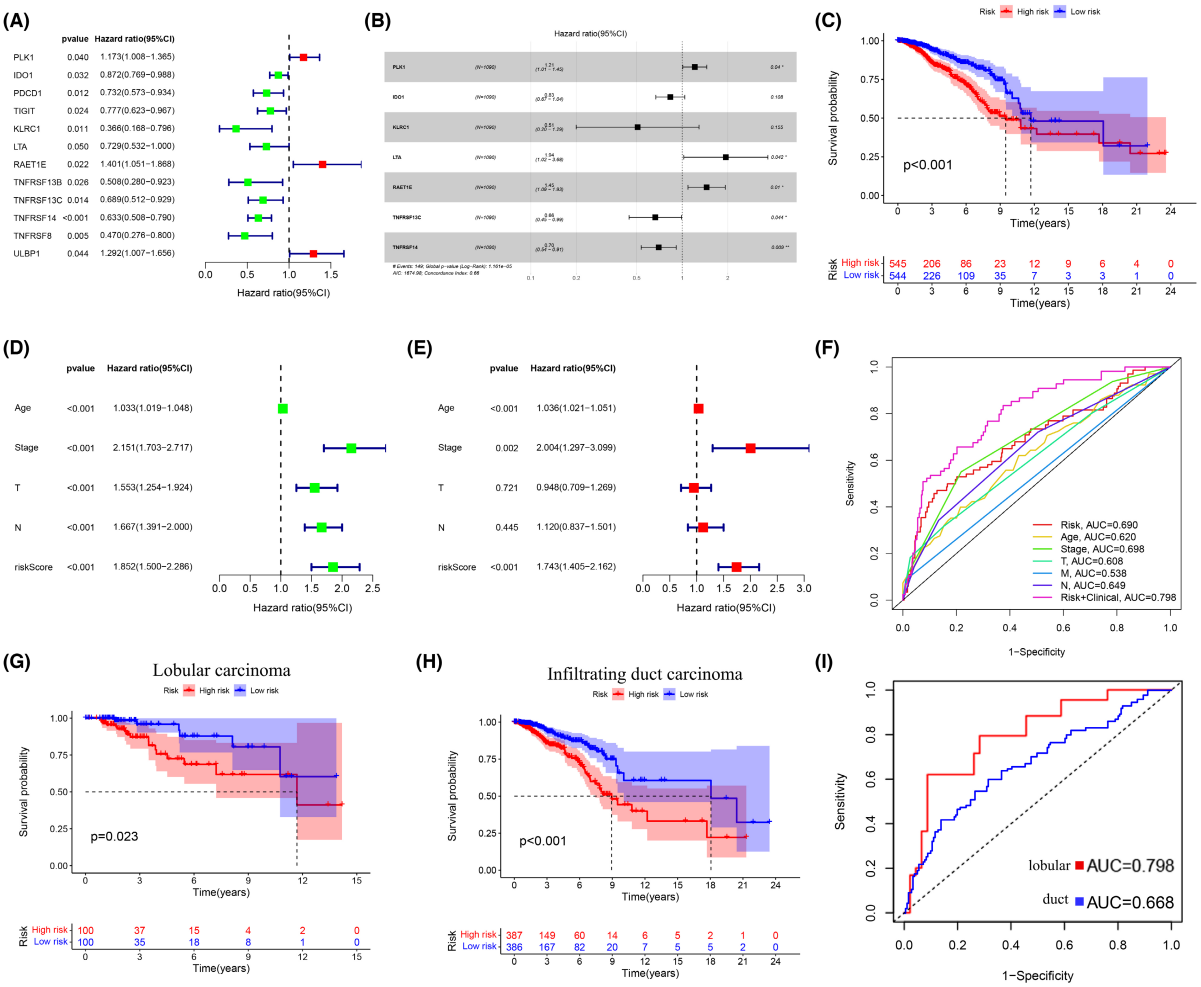
Establishment of a polo‐like kinase 1 (PLK1)‐based immune risk model for breast cancer (BRCA) prognosis prediction. The forest maps revealing the risk ratio of (A) 12 immunomodulatory factors associated with BRCA prognosis and (B) seven genes associated with PLK1 expression in BRCA for prognosis model construction. (C) Kaplan–Meier analysis of the overall survival between high‐ and low‐risk BRCA patients stratified by the PLK1‐based immune risk model. (D, E) Forest maps comparing the risk model with clinical features using univariate and multivariate Cox regression analysis, indicating the independence of this model in prognosis prediction. (F) The time‐dependent receiver operating characteristic (ROC) curve reveals satisfactory accuracy of the risk model in prognosis prediction compared to conventional clinical factors. (G–I) Survival curves and ROC curves based on the PLK1‐based immunological risk model in infiltrating duct carcinoma and lobular carcinoma.

**FIGURE 9:**
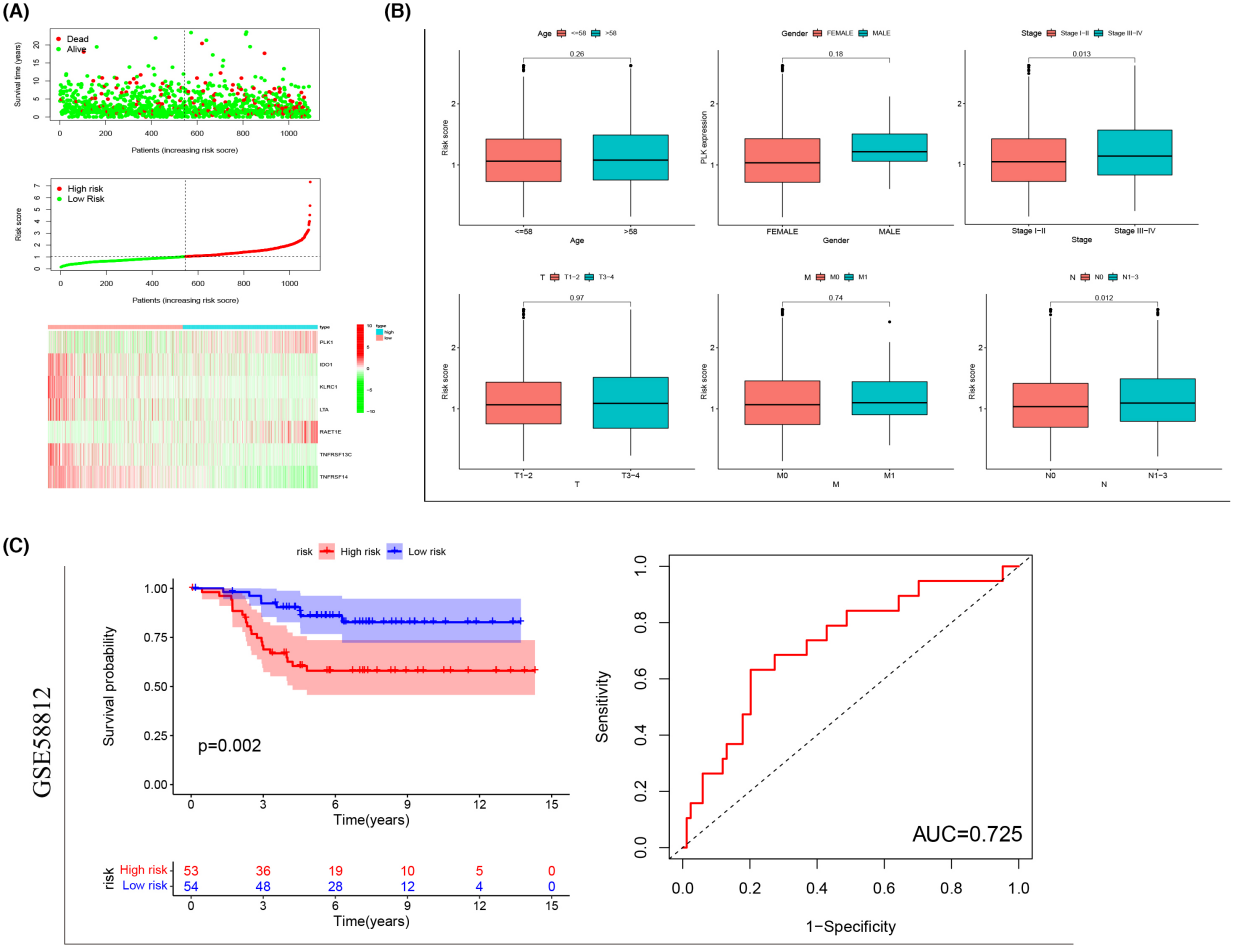
Validation of the polo‐like kinase 1 (PLK1)‐based immune risk model. (A) Comparisons of the overall survival and gene expression profiles between high‐ and low‐risk breast cancer patients stratified by the risk model. (B) Age, sex, and clinical stage (particularly the N stage) are significantly correlated with the risk model. (C) Survival curves and receiver operating characteristic curves based on the PLK1‐based immunological risk model in GSE58812 dataset.

Considering the direct or indirect susceptibility of a single gene to environmental factors, a multiple‐gene signature was considered for a stable prediction. The univariate Cox regression analysis revealed 12 immunomodulators most related to BRCA prognosis, and their relevancy was shown using the forest map (Figure 8A). Thereafter, seven genes (PLK1, IDO1, KLRC1, LTA, RAE1E, TNFRSF13C, and TNFRSF14) were confirmed by the multivariate Cox regression analysis (Figure 8B). The risk score of each patient was calculated based on the expressions of these genes as follows: risk score = PLK1 × 0.1916 + IDO1 × (−0.1807) + KLRC1 × (−0.6780) + LTA × 0.6634 + RAET1E × 0.3713 + TNFRSF13C × (−0.4095) + TNFRSF14 × (−0.3574). All BRCA patients from TCGA were assigned to the high‐ (*n* = 545) or low‐risk (*n* = 545) group based on the risk score, and the prediction performance of the model was assessed. The KM survival analysis revealed a shorter OS of the high‐risk patients compared to the low‐risk patients (*p* < 0.001) (Figure 8C). Additionally, univariate and multivariate Cox regression analysis were used to compare the risk model to clinical characteristics, and the findings suggested that this model is independent of clinical features in predicting prognosis (Figure 8D,E). Furthermore, as evidenced by ROC curves, this model demonstrated acceptable accuracy, which was considerably greater than PLK1 overexpression (AUC values: 0.690 vs. 0.649; Figure 8F). AUC of 0.798 was observed when the risk model was merged with clinical characteristics such as age, clinical stage, and TNM stage, improving the performance of the combined model in prognosis prediction. Additionally, to achieve more effective predictive performance, we further defined the PLK1‐associated model as having a survival significance consistent with the above model in both lobular and infiltrating duct carcinoma (Figure 8G,H), with an AUC value of 0.798 in lobular carcinoma (Figure 8I) indicating higher sensitivity. The risk curve showed higher mortality in high‐risk patients compared to the low‐risk patients (Figure 9A). Intriguingly, our model also had associations with the clinical stage (*p* = 0.013), particularly the N stage (*p* = 0.012), of the high‐risk BRCA patients (Figure 9B). Our results ascertained a good performance of the PLK1‐based immune risk model in predicting BRCA progression. Lastly, the PLK1‐based immune risk model was evaluated using the TNBC dataset GSE58812 and demonstrated consistency (*p* = 0.002, AUC = 0.725; Figure 9C).

**FIGURE 10:**
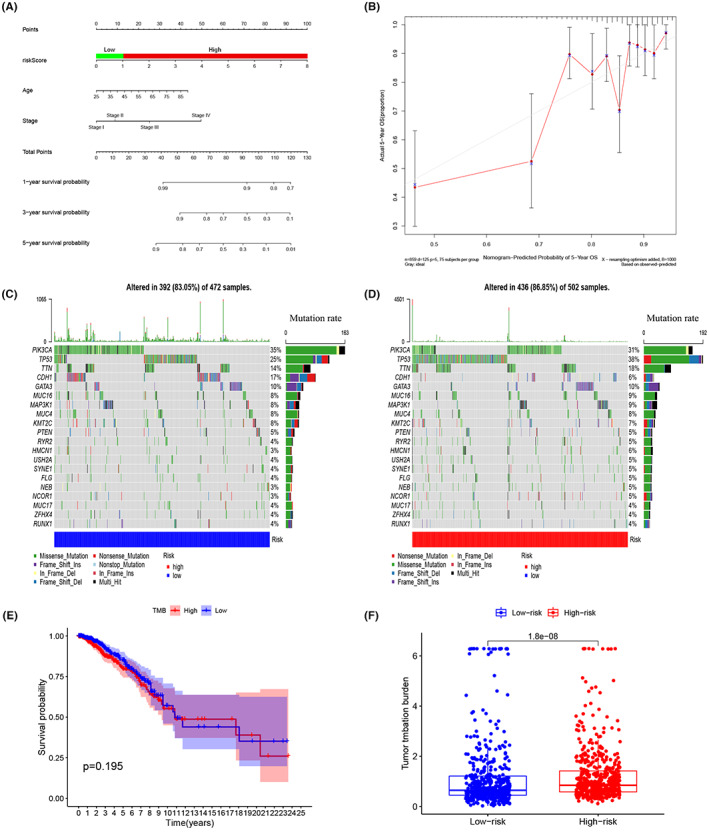
Nomogram construction, gene mutation prediction, and tumor mutation burden (TMB) estimation using the polo‐like kinase 1 (PLK1)‐based immune risk model. (A, B) Nomogram and calibration curves for predicting 1‐, 3‐, and 5‐year survival of patients with breast cancer. (C, D) Gene mutation waterfall maps organized by the risk score. (E) No difference was observed between TMB and patient prognosis, as evidenced by the Kaplan–Meier survival curve. (F) Difference in TMB between high‐ and low‐risk patients, as determined by the differential analysis.

On the basis of the total score, a predictive nomogram integrating the risk score, age, and clinical stage was built to estimate the 1‐, 3‐, and 5‐year survival probability of BRCA patients (Figure 10A). The calibration curve revealed a minor discrepancy between the nomogram's predicted 5‐year survival rate (gray line) and the actual 5‐year survival rate (red line) of the patients (Figure 10B). As a result, the prognostic nomogram demonstrated a high degree of predictive potential for BRCA survival.

### 3.6 Mutated genes and TMB in BRCA patients

The gene mutation profiles of BRCA patients were also evaluated using the risk model. It was shown that the PI3KCA, TP53, and TTN genes have common missense mutations in the patients. Among these, TP53 and TTN mutation rates were observed in the high‐risk group, while PI3KCA and CDH1 mutation rates were observed in the low‐risk group (Figure 10C,D). In contrast, TMB demonstrated no correlation with patient prognosis (*p* = 0.195; Figure 10E). However, high‐risk BRCA individuals had a significantly higher TMB value (*p* < 0.001; Figure 10F). The raw data of TMB for the selected BRCA cases are shown in Table S1.

### 3.7 The PLK1‐based immune risk model predicts immunotherapy and chemotherapy response of BRCA patients

**FIGURE 11:**
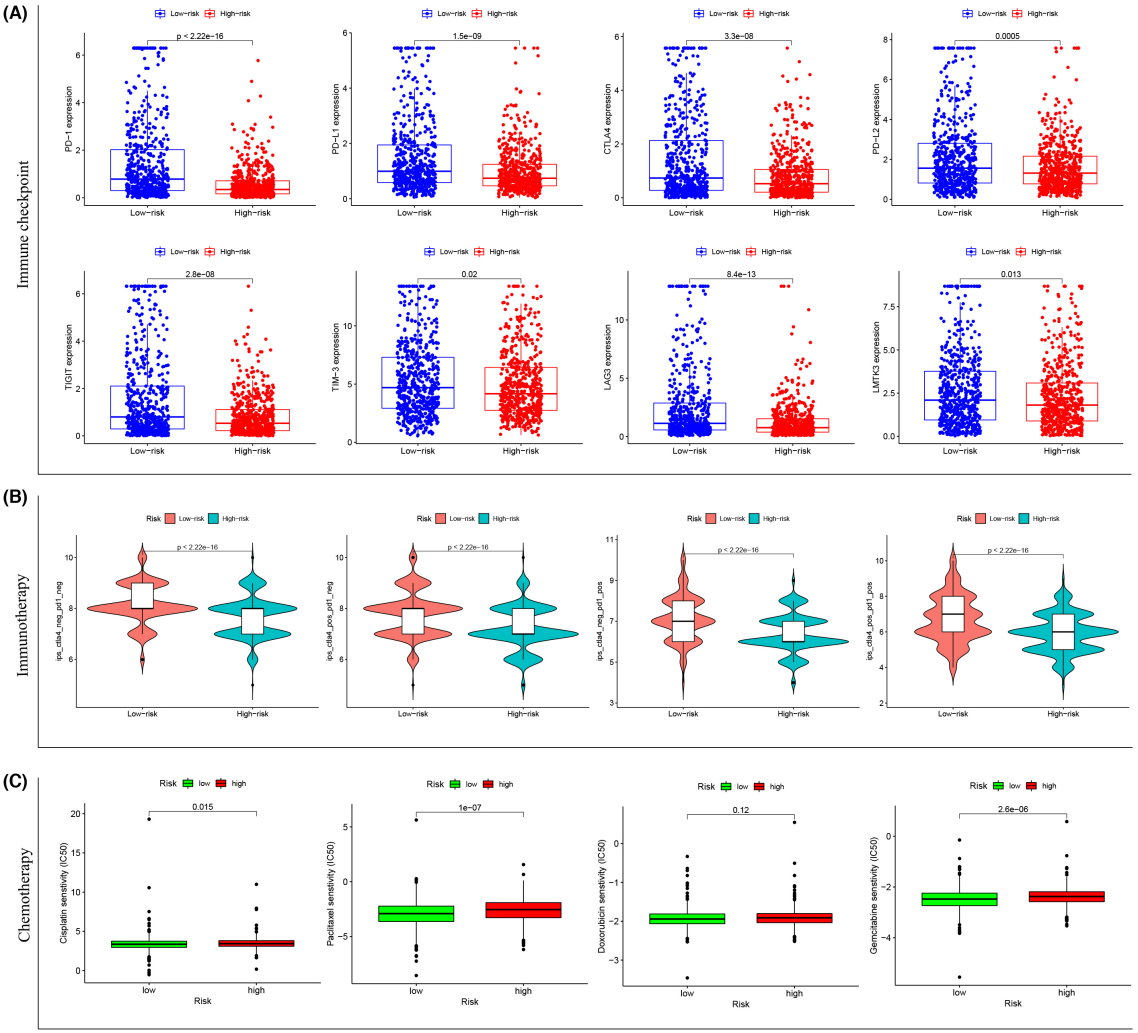
The polo‐like kinase 1 (PLK1)‐based immune risk model predicts immunotherapy and chemotherapy response of breast cancer (BRCA) patients. (A) Eight common immune checkpoints (PD‐1, PD‐L1, PD‐L2, CTLA4, LAG3, TIM‐3, TIGIT, and LMTK3) significantly associated with the PLK1‐based immune risk model in BRCA. (B) The immunotherapy analysis, based on the risk score, reveals an insufficient response to anti‐CTLA4 and/or anti‐PD1 drugs in high‐risk BRCA patients. (C) Sensitivity analysis of four common chemotherapeutic drugs (cisplatin, paclitaxel, doxorubicin, and gemcitabine) using the risk model.

We carried out the assessment for immunotherapy response prediction using the risk model and eight immune checkpoints (PD‐1, PD‐L1, PD‐L2, CTLA4, LAG3, TIM‐3, TIGIT, and LMTK3), which were significantly upregulated in low‐risk patients (Figure 11A). The immunotherapy analysis revealed an insufficient response to anti‐CTLA4 and/or anti‐PD1 drugs in high‐risk BRCA patients (Figure 11B). For chemotherapy response, the drug sensitivity analysis based on IC_50_ values showed a good response to paclitaxel, cisplatin, and gemcitabine in low‐risk patients compared to the high‐risk patients; however, there was no significant difference in patient response to doxorubicin (Figure 11C). These findings suggest that our model can serve as a useful tool for guiding therapeutic decision‐making for BRCA patients.

## 4 DISCUSSION

The immune system has a critical role in the onset and progression of BRCA. Immune cell infiltration, particularly of CD4^+^ or CD8^+^ T cell, B cell, monocyte, macrophage, dendritic cell, and NK cell infiltration, develops in lockstep with progressive cancerization of breast tissues.^22^, ^23^, ^24^ Several studies have demonstrated that TIL levels have tight associations with prognosis and improvements in adjuvant or neoadjuvant treatment response of BRCA patients, with higher TIL levels representing a lower recurrence and mortality risk.^25^, ^26^, ^27^ However, different subtypes of TILs have different functions. CD8^+^ T and T‐bet^+^ cells are indicators of a better prognosis or disease‐free survival,^28^, ^29^ while FOXP3‐positive TILs like Tregs often indicate poor OS and a higher risk of recurrence.^30^ Different immunomodulatory factors can stimulate an anti‐tumor immune response or restrain tumor immune escape during carcinogenesis or tumor progression. Immune checkpoints have been extensively studied in clinical and basic research, particularly PD‐L1, whose expression is associated with poor differentiation and prognosis in BRCA.^31^ However, when paired with chemotherapy, the PD‐L1 inhibitor, atezolizumab, has shown significant effectiveness in advanced TNBC.^8^, ^32^

Patients with advanced BRCA have poor prognosis and limited treatment options. The use of immunotherapy, especially immune checkpoint inhibitors (ICIs) combined with chemotherapy or targeted drugs can be an effective treatment for these patients; however, the overall response to ICI is insufficient.^33^, ^34^ Therefore, the first priority is to identify patients who may benefit from ICI therapy by analyzing their TIME conditions before the detection of any signs of tumor development, as this may aid in appropriate therapeutic decision‐making. However, the absence of reliable biomarkers for predicting BRCA prognosis, TIL distribution, and immunotherapy or chemotherapy response impede the resolution of this issue. Our study aimed to develop an immunological risk model based on PLK1 expression that could account for the aforementioned factors. PLK1 is involved in a variety of tumorogenic processes, including carcinogenesis, proliferation, EMT, and chemoresistance.^13^, ^14^, ^15^, ^16^ PLK1 is also involved in the regulation of NF‐κB signaling pathway, which is crucial for the immunological response or inflammation in the body.^13^, ^35^ Therefore, PLK1 is a promising biomarker for TIME assessment in BRCA patients. First, we confirmed that PLK1 overexpression is associated with shorter OS in BRCA patients. Although the survival curves intersected, we noted that in the 20‐year follow‐up of the BRCA cohort, which included 1090 people, the number of persons with 8 years of follow‐up (102) was <10%, and the number of persons with 10 years of follow‐up (40) was <5%. As a result, we concluded that a cohort with such a minimal number of observations already has a significant bias. The results indicated that during the ninth year, the survival rate of the otherwise smooth low‐PLK1 expressing group decreased abruptly (with three deaths recorded in the low‐expression group and one death recorded in the high‐expression group) and intersected for the first time with the curve of the high‐expression groups, which was interpreted as a chance result due to the small sample size. Additionally, the findings were confirmed in a wider queue. Increased PLK1 expression was also associated with clinical stage, particularly stage IV BRCA. These data suggest an oncogenic role of PLK1 overexpression in BRCA, which promotes tumor initiation and progression and predicts a poor prognosis. Our results corroborate those obtained in previous investigations of PLK1 expression in malignant tumors.^12^, ^17^ Additionally, we observed a differential expression of PLK1 across BRCA subtypes, with an exceptionally high level of expression in basal‐like BRCA, suggesting that PLK1 may be a therapeutic target in these individuals.

Comparison of the BRCA tissues with the normal breast tissues revealed substantial shifts in the levels of various TILs, including CD8^+^ T cells, NK cells, Tregs, and dendritic cells, across time. There is a possibility that these TILs are directly involved in the emergence and evolution of BRCA. However, studies have revealed no link between PLK1 expression and immune cell infiltration. This study has identified 14 types of immune cells in BRCA, including Tregs,^36^ which have been shown to assist in the creation of an inhibitory microenvironment and demonstrated to be positively associated with PLK1 expression in BRCA. NK cells are responsible for tumor monitoring and destruction, whereas M2 macrophages often promote tumor development and dissemination.^37^ According to the TISIDB study, PLK1 expression is positively correlated with the infiltration of activated CD8^+^ T cells, which have a substantial anti‐tumor role. However, the associations between PLK1 and immune cell infiltration indicated above are similar to those found in the TISIDB analysis. Therefore, there are two lines of evidence supporting the role of PLK1 in both immunosuppression in TIME and the anti‐tumor immune response. A high enrichment of the PLK1 gene in T cell receptors, B cell receptors, or molecules of the chemokine signaling pathway was found using GSEA enrichment analysis, demonstrating that PLK1 is involved in controlling the immunity against BRCA via the regulation of immune cell infiltration. Further investigations are required to confirm the regulatory mechanisms of PLK1 in immune cell infiltration.

This was the first study to demonstrate that PLK1 was associated with a wide range of immunostimulators (e.g., CD80 and CD86) and immunoinhibitors (e.g., PD‐1, PD‐L1, and CTLA4). However, PPI networks revealed a very complex protein network between these components, making it impossible to discern the regulatory functions of PLK1 in tumor immunostimulation and immunosuppression. However, enrichment analysis revealed that immunomodulatory variables associated with PLK1 were mostly engaged in cellular response to stimuli, protein binding, and cytokine–cytokine receptor interactions. This finding suggested that PLK1 was extensively involved in TIME regulation in BRCA. We identified seven genes of PLK1‐associated immunomodulators (PLK1, IDO1, KLRC1, LTA, RAE1E, TNFRSF13C, and TNFRSF14) for construction of a Cox regression model, which was expressed by a hazard function. Subsequently, survival analysis and ROC curves exhibited an excellent performance in predicting BRCA prognosis, suggesting that they might be used as independent prognostic factors for BRCA survival prediction. Although clinical stage was more predictive than signature in the first 3 years, the AUC value of signature surpassed that of clinical stage after 3 years, showing that it has greater long‐term predictive importance. More importantly, the risk model demonstrated outstanding predictive performance for lobular carcinoma, which is predicted to result in more effective treatment implications for lobular carcinoma patients. Furthermore, the model with integrated clinical characteristics had even greater prediction accuracy. In comparison to low‐risk individuals, high‐risk individuals may have more objective signs, such as clinical stage and lymph node involvement. This data implies a more rapid clinical development in high‐risk BRCA individuals, emphasizing the vital need of early intervention for treatment effectiveness. Additionally, we developed a prognostic nomogram for estimating 1‐, 3‐, and 5‐year survival rates, making the risk model prediction accessible.

Missense mutations are the most common type of mutations in BRCA, followed by frameshift and nonsense mutations,^38^ and prior investigations have regularly revealed PIK3CA, TP53, GATA3, and CDH1 mutations in BRCA.^39^, ^40^ To understand the immunological characteristics of our risk model at the gene level, we compared the difference in gene mutations between the high and low‐risk groups. Our results revealed that PIK3CA, TP53, TTN, CDH1, and GATA3 mutations were most frequent (>10%) in BRCA, in which missense mutations are common. Among them, the probability of TP53 mutation was exceptionally high in high‐risk patients. TP53 is a key tumor suppressor gene, and TP53 expression in noncancerous tissues is closely associated with cell‐cycle arrest, DNA repair, and apoptosis, all of which contribute to the acceleration of tumor incidence and progression. There is evidence that the TP53 mutation confers a poor prognosis for BRCA^41^ patients, which is consistent with our survival data.

Additionally, we tested the predictive ability of the model for immunotherapy response. Anti‐PD‐1 and/or anti‐CTLA4 drugs had a substantial advantage on the low‐risk BRCA patients identified by our algorithm. Immune checkpoint expression, especially PD‐L1, is a useful biomarker for monitoring ICI therapy,^42^ and there is evidence that PD‐L1 positive BRCA individuals respond well to ICI treatment.^8^, ^32^ In this study, eight immune checkpoints, including PD‐1, PD‐L1/PD‐L2, and CTLA4, were observed to be significantly upregulated in the low‐risk patients, indicating a potential association between PD‐L1 expression and ICI treatment benefits in BRCA, as reported previously. TMB is a critical biomarker for identifying cancer patients who may benefit from ICI treatment because it increases tumor immunogenicity primarily by increasing the amount of mutant proteins that generate novel antigenic peptides.^43^, ^44^ In this study, we evaluated the overall prognostic significance of TMB before establishing its predictive value for ICI treatment. We found that TMB had no significant effect on the OS, excluding the interference from TMB, which can be used as an indicator for ICI treatment efficacy. A cohort study involving 8207 patients with various tumor types, including bladder cancer, non‐small cell lung cancer, melanoma, etc., showed that high TMB was associated with prolonged OS after anti‐PD‐1/PD‐L1 and/or anti‐CTLA4 treatment, and the treatment benefits increased with the increase in TMB value.^45^ Moreover, a study on TMB in BRCA patients, in conjunction with other latest evidence, shows that high TMB is also related to longer progression‐free survival following anti‐PD‐1/PD‐L1 treatment.^46^ However, it was shown that following immunotherapy, there was no discernible difference in the survival of BRCA patients with varying TMB levels.^45^ We observed a greater TMB level in patients with a high‐risk score, but the usage and research of TMB in BRCA are presently inconclusive for a variety of reasons. For instance, BRCA is a less immunogenic tumor, and TMB levels are often lower in BC,^47^ indicating an inadequate specificity for predicting immunotherapy benefits. Therefore, further study is required to establish the importance of TMB in BC.

For BRCA treatment, majority of doctors recommend chemotherapy, particularly in advanced patients. To assess the model's capacity to predict the effectiveness of chemotherapy, we compared the IC_50_ values of four commonly used BRCA chemotherapeutic agents in high‐ and low‐risk individuals. Paclitaxel, cisplatin, and gemcitabine had considerably lower IC_50_ values in the low‐risk group, suggesting that this group had a greater sensitivity to these agents. In general, the risk model successfully identified low‐risk populations that responded well to either common immunotherapy agents or chemotherapy drugs, indicating that it is a promising biomarker and a cost‐effective method of providing early prognosis prediction and response guidance for individual patients.

In summary, PLK1 upregulation is essential in TIME regulation in BRCA and facilitates in the determination of TIL distribution and immune regulating factors expression in BRCA patients. The immunological risk model we developed on the basis of PLK1 is capable of effectively predicting patient prognosis, tumor growth, the extent of immunotherapy and chemotherapy response, and identifying gene alterations. Its predictive ability may be enhanced by including clinical characteristics. The related nomogram graphically depicts the survival prediction data, making it practical and useful for BRCA therapy. In clinical practice, refractory BRCA and TNBC pose many challenges in the determination of treatment strategies, and our research provides a novel strategy for BRCA treatment, suggesting the use PLK1 inhibitors in combination with ICIs. We expect that this novel treatment strategy can significantly improve the prognosis of BRCA patients.

## AUTHOR CONTRIBUTIONS

**Yan Chen:** Resources (equal); validation (equal). **Yiqing You:** Software (equal); writing – original draft (lead). **Qiaoling Wu:** Data curation (equal); software (equal). **Jing Wu:** Formal analysis (equal); validation (equal). **Shujing Lin:** Validation (equal). **Yang Sun:** Conceptualization (equal); supervision (equal); writing – review and editing (equal). **Zhaolei Cui:** Conceptualization (equal); funding acquisition (equal); writing – review and editing (equal).

## FUNDING INFORMATION

This study was supported by the Provincial Natural Science Fund of Fujian (Grant number: 2021J01444), and Joint Funds for the Innovation of Science and Technology, Fujian province (Grant number: 2021Y9222), and The First Batch of High‐level Talent Training Program of Fujian Cancer Hospital.

## CONFLICT OF INTEREST STATEMENT

The authors declare that they have no competing interests.

## ETHICS STATEMENT

This article does not contain any studies with human participants or animals performed by any of the authors, therefore no ethic approval or consent is required. No administrative permission and/or licenses is acquired by this study to access the original data used in this research.

## Supporting information


Supplementary Table 1.

## Data Availability

The data that support the findings of this study are available on request from the corresponding author.

## References

[1] Bray F , Ferlay J , Soerjomataram I , Siegel RL , Torre LA , Jemal A . Global cancer statistics 2018: GLOBOCAN estimates of incidence and mortality worldwide for 36 cancers in 185 countries. CA Cancer J Clin. 2018;68(6):394‐424. 30207593 10.3322/caac.21492

[2] Waks AG , Winer EP . Breast cancer treatment: a review. JAMA. 2019;321(3):288‐300. 30667505 10.1001/jama.2018.19323

[3] Garrido‐Castro AC , Lin NU , Polyak K . Insights into molecular classifications of triple‐negative breast cancer: improving patient selection for treatment. Cancer Discov. 2019;9(2):176‐198. 30679171 10.1158/2159-8290.CD-18-1177 PMC6387871

[4] Ammannagari N , Atasoy A . Current status of immunotherapy and immune biomarkers in gastro‐esophageal cancers. J Gastrointest Oncol. 2018;9(1):196‐207. 29564185 10.21037/jgo.2017.06.12 PMC5848035

[5] Yang S , Zhang Z , Wang Q . Emerging therapies for small cell lung cancer. J Hematol Oncol. 2019;12(1):47. 31046803 10.1186/s13045-019-0736-3 PMC6498593

[6] Thoma C . Kidney cancer: combining targeted and immunotherapy. Nat Rev Urol. 2018;15(5):263. 10.1038/nrurol.2018.43 29620060

[7] de Melo GD , Buzaid AC , Perez‐Garcia J , Cortes J . Immunotherapy in breast cancer: current practice and clinical challenges. BioDrugs. 2020;34(5):611‐623. 32870473 10.1007/s40259-020-00436-9

[8] Schmid P , Adams S , Rugo HS , et al. Atezolizumab and nab‐paclitaxel in advanced triple‐negative breast cancer. N Engl J Med. 2018;379(22):2108‐2121. 30345906 10.1056/NEJMoa1809615

[9] van de Weerdt BC , Medema RH . Polo‐like kinases: a team in control of the division. Cell Cycle. 2006;5(8):853‐864. 16627997 10.4161/cc.5.8.2692

[10] Wang ZX , Xue D , Liu ZL , et al. Overexpression of polo‐like kinase 1 and its clinical significance in human non‐small cell lung cancer. Int J Biochem Cell Biol. 2012;44(1):200‐210. 22064247 10.1016/j.biocel.2011.10.017

[11] Seyedabadi S , Saidijam M , Najafi R , et al. Assessment of CEP55, PLK1 and FOXM1 expression in patients with bladder cancer in comparison with healthy individuals. Cancer Invest. 2018;36(8):407‐414. 30277841 10.1080/07357907.2018.1514504

[12] Gheghiani L , Wang L , Zhang Y , et al. PLK1 induces chromosomal instability and overrides cell‐cycle checkpoints to drive tumorigenesis. Cancer Res. 2021;81(5):1293‐1307. 33376114 10.1158/0008-5472.CAN-20-1377 PMC8026515

[13] Iliaki S , Beyaert R , Afonina IS . Polo‐like kinase 1 (PLK1) signaling in cancer and beyond. Biochem Pharmacol. 2021;193:114747. 34454931 10.1016/j.bcp.2021.114747

[14] Wu J , Ivanov AI , Fisher PB , Fu Z . Polo‐like kinase 1 induces epithelial‐to‐mesenchymal transition and promotes epithelial cell motility by activating CRAF/ERK signaling. Elife. 2016;5:e10734. 27003818 10.7554/eLife.10734 PMC4811775

[15] Matthess Y , Raab M , Knecht R , Becker S , Strebhardt K . Sequential Cdk1 and PlK1 phosphorylation of caspase‐8 triggers apoptotic cell death during mitosis. Mol Oncol. 2014;8:596‐608. 24484936 10.1016/j.molonc.2013.12.013 PMC5528627

[16] Gutteridge RE , Ndiaye MA , Liu X , Ahmad N . Plk1 inhibitors in cancer therapy: from laboratory to clinics. Mol Cancer Ther. 2016;15:1427‐1435. 27330107 10.1158/1535-7163.MCT-15-0897 PMC4936921

[17] Liu Z , Sun Q , Wang X . PLK1, a potential target for cancer therapy. Transl Oncol. 2017;10:22‐32. 27888710 10.1016/j.tranon.2016.10.003 PMC5124362

[18] Elsayed I , Wang X . PlK1 inhibition in cancer therapy: potentials and challenges. Future Med Chem. 2019;11(12):1383‐1386. 31298578 10.4155/fmc-2019-0084

[19] Newman AM , Liu CL , Green MR , et al. Robust enumeration of cell subsets from tissue expression profiles. Nat Methods. 2015;12(5):453‐457. 25822800 10.1038/nmeth.3337 PMC4739640

[20] Geeleher P , Cox N , Huang RS . pRRophetic: an R package for prediction of clinical chemotherapeutic response from tumor gene expression levels. PLoS ONE. 2014;9(9):e107468. 25229481 10.1371/journal.pone.0107468 PMC4167990

[21] Weigelt B , Baehner FL , Reis‐Filho JS . The contribution of gene expression profiling to breast cancer classification, prognostication and prediction: a retrospective of the last decade. J Pathol. 2010;220(2):263‐280. 19927298 10.1002/path.2648

[22] Azizi E , Carr AJ , Plitas G , et al. Single‐cell map of diverse immune phenotypes in the breast tumor microenvironment. Cell. 2018;174(5):1293‐1308.e1236. 29961579 10.1016/j.cell.2018.05.060 PMC6348010

[23] Kim M , Chung YR , Kim HJ , Woo JW , Ahn S , Park SY . Immune microenvironment in ductal carcinoma in situ: a comparison with invasive carcinoma of the breast. Breast Cancer Res. 2020;22(1):32. 32216826 10.1186/s13058-020-01267-w PMC7098119

[24] Chen XY , Yeong J , Thike AA , Bay BH , Tan PH . Prognostic role of immune infiltrates in breast ductal carcinoma in situ. Breast Cancer Res Treat. 2019;177(1):17‐27. 31134489 10.1007/s10549-019-05272-2

[25] Adams S , Gray RJ , Demaria S , et al. Prognostic value of tumor‐infiltrating lymphocytes in triple‐negative breast cancers from two phase III randomized adjuvant breast cancer trials: ECOG 2197 and ECOG 1199. J Clin Oncol. 2014;32(27):2959‐2966. 25071121 10.1200/JCO.2013.55.0491 PMC4162494

[26] Savas P , Salgado R , Denkert C , et al. Clinical relevance of host immunity in breast cancer: from TILs to the clinic. Nat Rev Clin Oncol. 2016;13(4):228‐241. 26667975 10.1038/nrclinonc.2015.215

[27] Loi S , Michiels S , Salgado R , et al. Tumor infiltrating lymphocytes are prognostic in triple negative breast cancer and predictive for trastuzumab benefit in early breast cancer: results from the FinHER trial. Ann Oncol. 2014;25(8):1544‐1550. 24608200 10.1093/annonc/mdu112

[28] Mahmoud SM , Paish EC , Powe DG , et al. Tumor‐infiltrating CD8+ lymphocytes predict clinical outcome in breast cancer. J Clin Oncol. 2011;29(15):1949‐1955. 21483002 10.1200/JCO.2010.30.5037

[29] Mulligan AM , Pinnaduwage D , Tchatchou S , Bull SB , Andrulis IL . Validation of intratumoral T‐bet+ lymphoid cells as predictors of disease‐free survival in breast cancer. Cancer Immunol Res. 2016;4(1):41‐48. 26546451 10.1158/2326-6066.CIR-15-0051

[30] Bates GJ , Fox SB , Han C , et al. Quantification of regulatory T cells enables the identification of high‐risk breast cancer patients and those at risk of late relapse. J Clin Oncol. 2006;24(34):5373‐5380. 17135638 10.1200/JCO.2006.05.9584

[31] Huang W , Ran R , Shao B , Li H . Prognostic and clinicopathological value of PD‐L1 expression in primary breast cancer: a meta‐analysis. Breast Cancer Res Treat. 2019;178(1):17‐33. 31359214 10.1007/s10549-019-05371-0

[32] Schmid P , Rugo HS , Adams S , et al. Atezolizumab plus nab‐paclitaxel as first‐line treatment for unresectable, locally advanced or metastatic triple‐negative breast cancer (IMpassion130): updated efficacy results from a randomised, double‐blind, placebo‐controlled, phase 3 trial. Lancet Oncol. 2020;21(1):44‐59. 31786121 10.1016/S1470-2045(19)30689-8

[33] Cortes J , Cescon DW , Rugo HS , et al. Pembrolizumab plus chemotherapy versus placebo plus chemotherapy for previously untreated locally recurrent inoperable or metastatic triple‐negative breast cancer (KEYNOTE‐355): a randomised, placebo‐controlled, double‐blind, phase 3 clinical trial. Lancet. 2020;396(10265):1817‐1828. 33278935 10.1016/S0140-6736(20)32531-9

[34] Liu J , Blake SJ , Yong MC , et al. Improved efficacy of neoadjuvant compared to adjuvant immunotherapy to eradicate metastatic disease. Cancer Discov. 2016;6(12):1382‐1399. 27663893 10.1158/2159-8290.CD-16-0577

[35] Mitchell S , Vargas J , Hoffmann A . Signaling via the NFκB system. Wiley interdisciplinary reviews. Wiley Interdiscip Rev Syst Biol Med. 2016;8:227‐241. 26990581 10.1002/wsbm.1331 PMC8363188

[36] Wang H , Franco F , Ho PC . Metabolic regulation of Tregs in cancer: opportunities for immunotherapy. Trends Cancer. 2017;3(8):583‐592. 28780935 10.1016/j.trecan.2017.06.005

[37] Medrek C , Pontén F , Jirström K , Leandersson K . The presence of tumor associated macrophages in tumor stroma as a prognostic marker for breast cancer patients. BMC Cancer. 2012;12:306. 22824040 10.1186/1471-2407-12-306 PMC3414782

[38] Cancer Genome Atlas Network . Comprehensive molecular portraits of human breast tumours. Nature. 2012;490(7418):61‐70. 23000897 10.1038/nature11412 PMC3465532

[39] Freitag CE , Mei P , Wei L , Parwani AV , Li Z . Genetic alterations and their association with clinicopathologic characteristics in advanced breast carcinomas: focusing on clinically actionable genetic alterations. Hum Pathol. 2020;102:94‐103. 32445652 10.1016/j.humpath.2020.05.005

[40] Lefebvre C , Bachelot T , Filleron T , et al. Mutational profile of metastatic breast cancers: a retrospective analysis. PLoS Med. 2016;13(12):e1002201. 28027327 10.1371/journal.pmed.1002201 PMC5189935

[41] Olivier M , Langerød A , Carrieri P , et al. The clinical value of somatic TP53 gene mutations in 1,794 patients with breast cancer. Clin Cancer Res. 2006;12(4):1157‐1167. 16489069 10.1158/1078-0432.CCR-05-1029

[42] Gibney GT , Weiner LM , Atkins MB . Predictive biomarkers for checkpoint inhibitor‐based immunotherapy. Lancet Oncol. 2016;17(12):e542‐e551. 27924752 10.1016/S1470-2045(16)30406-5 PMC5702534

[43] Cristescu R , Mogg R , Ayers M , et al. Pan‐tumor genomic biomarkers for pd‐1 checkpoint blockade‐based immunotherapy. Science. 2018;362(6411):eaar3593. 30309915 10.1126/science.aar3593 PMC6718162

[44] Chan TA , Yarchoan M , Jaffee E , et al. Development of tumor mutation burden as an immunotherapy biomarker: utility for the oncology clinic. Ann Oncol. 2019;30:44‐56. 30395155 10.1093/annonc/mdy495 PMC6336005

[45] Samstein RM , Lee CH , Shoushtari AN , et al. Tumor mutational load predicts survival after immunotherapy across multiple cancer types. Nat Genet. 2019;51(2):202‐206. 30643254 10.1038/s41588-018-0312-8 PMC6365097

[46] Barroso‐Sousa R , Keenan TE , Pernas S , et al. Tumor mutational burden and PTEN alterations as molecular correlates of response to PD‐1/L1 blockade in metastatic triple‐negative breast cancer. Clin Cancer Res. 2020;26(11):2565‐2572. 32019858 10.1158/1078-0432.CCR-19-3507 PMC7269810

[47] Barroso‐Sousa R , Jain E , Cohen O , et al. Prevalence and mutational determinants of high tumor mutation burden in breast cancer. Ann Oncol. 2020;31(3):387‐394. 32067680 10.1016/j.annonc.2019.11.010

